# Author Correction: Multifunctional roles of Brl1-Brr6 in nuclear envelope fusion during nuclear pore complex biogenesis

**DOI:** 10.1038/s44318-026-00865-2

**Published:** 2026-07-20

**Authors:** Sayan Mondal, Annett Neuner, Azqa Ajmal Khan, Jlenia Vitale, Elmar Schiebel

**Affiliations:** 1 Zentrum für Molekulare Biologie der Universität Heidelberg (ZMBH), Deutsches Krebsforschungszentrum (DKFZ)-ZMBH Allianz, Universität Heidelberg, Heidelberg, 69120 Germany; 2 Heidelberg Biosciences International Graduate School (HBIGS), Universität Heidelberg, Heidelberg, 69120 Germany; 3 Present Address: EMBL, Meyerhofstraße 1, Heidelberg, 69177 Germany

## Abstract

**Figure:**
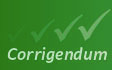

**Correction to:** *The EMBO Journal* (2026) 45:2370–2399. 10.1038/s44318-026-00718-y | Published online 16 February 2026

**A reference is added**.

**The Discussion section is corrected**.

The final paragraph of the Discussion is corrected from:

Homologs of Brl1 and Brr6 are found in organisms that retain the NE during mitosis suggesting that the Brl1/Brr6 NE fusion machinery is conserved (Tamm et al, 2011). Interestingly, a remote-homology search identified CLCC1 as a putative Brl1/Brr6 homolog with potential roles in NPC assembly (preprint: Mathiowetz et al, 2024). It will be intriguing to determine which functional domains within CLCC1 mediate Nup recruitment and INM/ONM fusion.

To: (changes in bold)

*Homologs of Brl1 and Brr6 are found in organisms that retain the NE during mitosis suggesting that the Brl1/Brr6 NE fusion machinery is conserved (Tamm et al, 2011). Interestingly, a remote-homology search identified CLCC1 as a putative Brl1/Brr6 homolog with potential roles in NPC assembly (preprint: Mathiowetz et al, 2024)*. **Furthermore, another study (preprint:** Fischer et al, 2025**) recently also used mutations flanking the PAL motif to reach similar conclusions regarding function and overexpression toxicity, and implicated CLCC1 as NPC fusogen in human and fly cells**.

It will be intriguing to determine which functional domains within CLCC1 mediate Nup recruitment and INM/ONM fusion.

One additional reference is added to the reference list:
