# Supplementary material for: Multifunctional roles of Brl1-Brr6 in nuclear envelope fusion during nuclear pore complex biogenesis
Source: EMBO J. 2026 Feb 16;45(7):2370–99. doi: 10.1038/s44318-026-00718-y (PMC13043894; doi:10.1038/s44318-026-00718-y)
Supplement: Supplementary file 1 — Appendix [file 44318_2026_718_MOESM1_ESM.pdf]

Appendix for

Multifunctional Roles of Brl1-Brr6 in Nuclear Envelope Fusion During Nuclear Pore Complex Biogenesis

Table of contents:

Appendix Figure S1 - Brl1 and Brr6 are predicted to form oligomers by AlphaFold.	2
Appendix Figure S2 - Phenotypes of cold sensitive *brr6*^{L145E} cells.	4
Appendix Figure S3 - Comparison of *S. cerevisiae* Brl1, Brr6 and *S. pombe* Brr6.	5
Appendix Figure S4 - Nsp1 immuno-EM of pGal1-*BRR6* and pGal1-*brr6*^{PAL} cells.	7
Appendix Figure S5 - *brl1*^{C343Y} mutant cells develop herniations.	9
Appendix Figure S6 - Analysis of the pGal1-*brl1*$^{ePNS\text{-}CA}$ and pGal1-*brr6*$^{ePNS\text{-}CA}$ phenotypes.	11

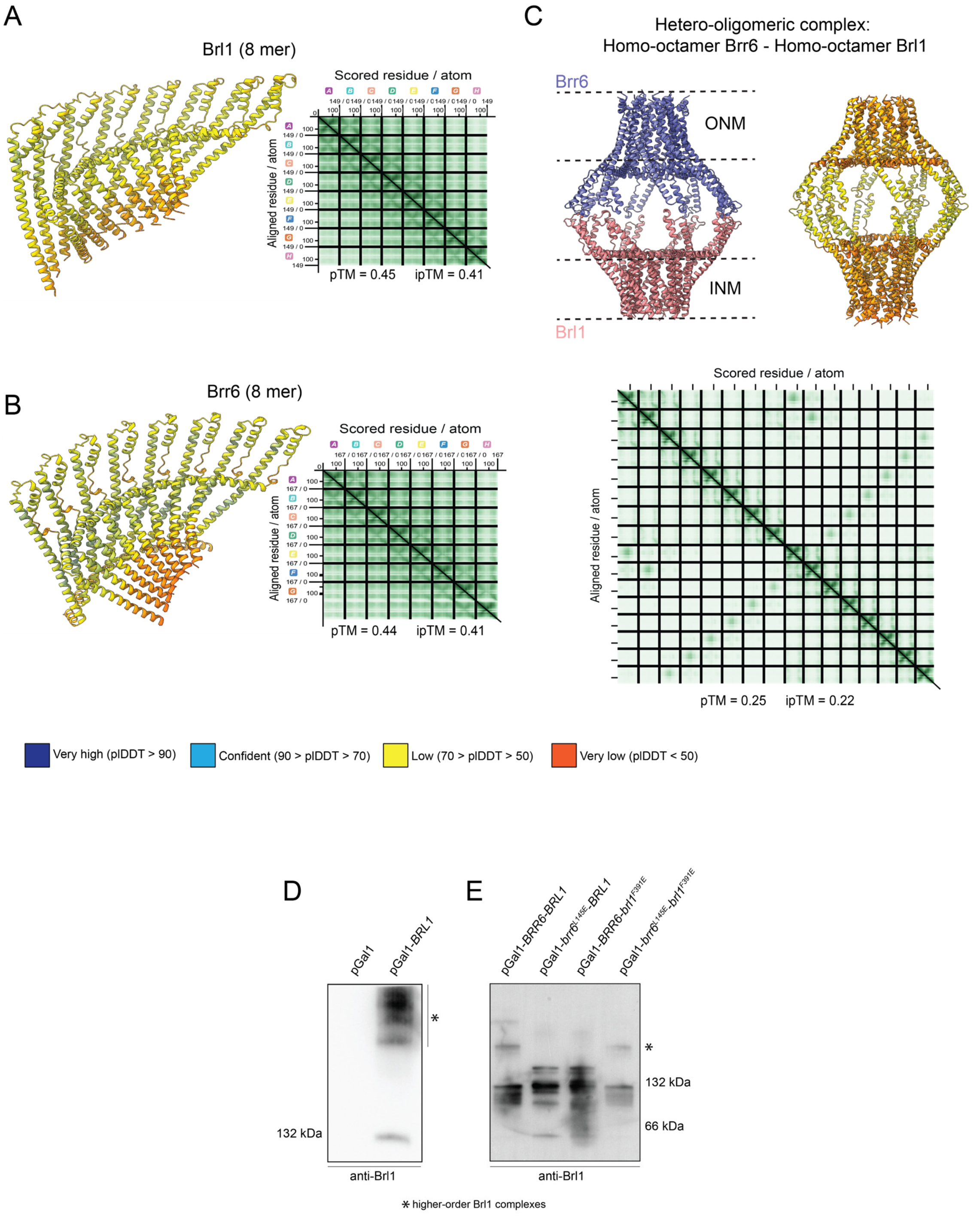

Appendix Figure S1 - Brl1 and Brr6 are predicted to form oligomers by AlphaFold. Extension of Fig. 1.

(A,B) AlphaFold predicts that eight Brl1 (A) or eight Brr6 (B) molecules form a sheet like assembly. (**C**) Predicated co-assembly of eight Brl1 and eight Brr6 molecules in a dome likely

structure that spans the intermembrane space. INM and ONM are indicated. Note, in this prediction Brl1 and Brr6 show head-to-head DAH interactions via the PAL sequences as shown in Fig. 1D. The structure on the right indicates the confidence score of the assembly. The color code is provided below. **(D)** Evidence for larger oligomeric Brl1 assemblies was obtained from gel analysis of strains expressing pGal1-*BRL1*. Brl1-containing complexes of approximately 130 kDa, along with higher molecular weight complexes (indicated by asterisk), were detected using Brl1-specific antibodies. (**E**) Mutations in the AαH of Brr6 (L145E) or Brl1 (F391E) abolished the formation of high molecular weight Brl1 complexes (indicated by asterisk). However, co-expression of $brr6^{L145E}$ and $brl1^{F391E}$ restore higher molecular weight Brl1 assemblies.

A

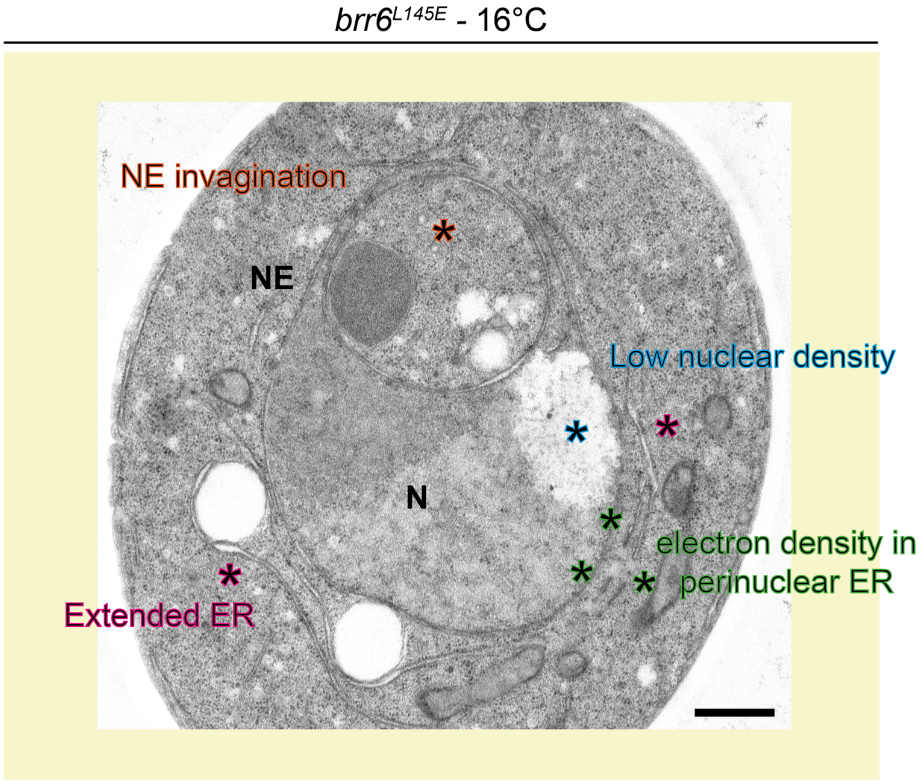

B

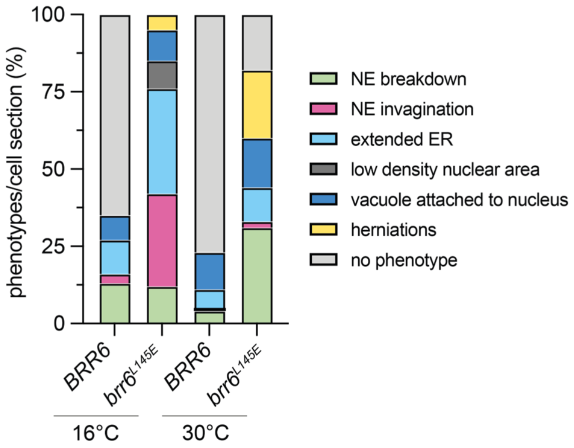

Appendix Figure S2 - Phenotypes of cold sensitive *brr6*^{L145E} cells. Extension of Fig. 4. **(A)** Summary of EM phenotypes in *brr6*^{L145E} cells incubated over night at 16ºC. Size bar: 200 nm. (**B)** Quantification of phenotypes observed in Fig. 4D. Cells were categorized as outlined in (A). *BRR6* (16°C): *n* = 57; *brr6*^{L145E} (16°C): *n* = 78; *BRR6* (30°C): *n* = 77; *brr6*^{L145E} (30°C): *n* = 67.

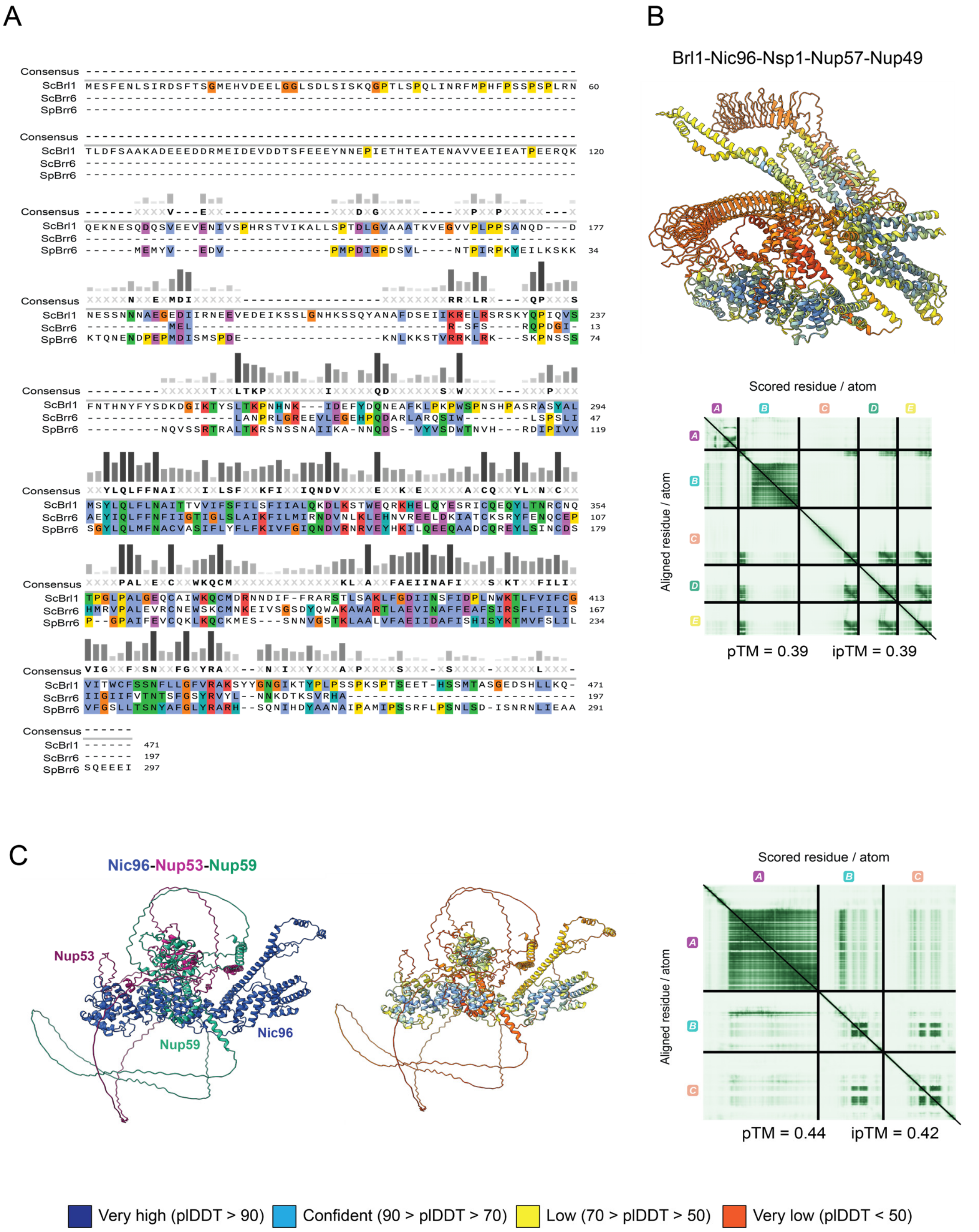

Appendix Figure S3 - Comparison of *S. cerevisiae* Brl1, Brr6 and *S. pombe* Brr6.

Extension of Fig. 6.

(A) Amino acid sequence alignment of Brl1 and Brr6 from *Saccharomyces cerevisiae* (ScBrl1 and ScBrr6) and Brr6 from *Schizosaccharomyces pombe* (SpBrr6). The alignment highlights

conservation not only within the DAH domain containing four cysteine residues, but also between the N-terminal regions of *S. cerevisiae* Brl1 *and S. pombe* Brr6. **(B)** Scores of AlphaFold prediction of the Brl1-Nic96-Nsp1-Nup57-Nup49 interaction. **(C)** The Brl1–Nic96 interface predicted in Fig. 6D is blocked in the Nic96 complex by Nup53 and Nup59 once incorporated into the NPC.

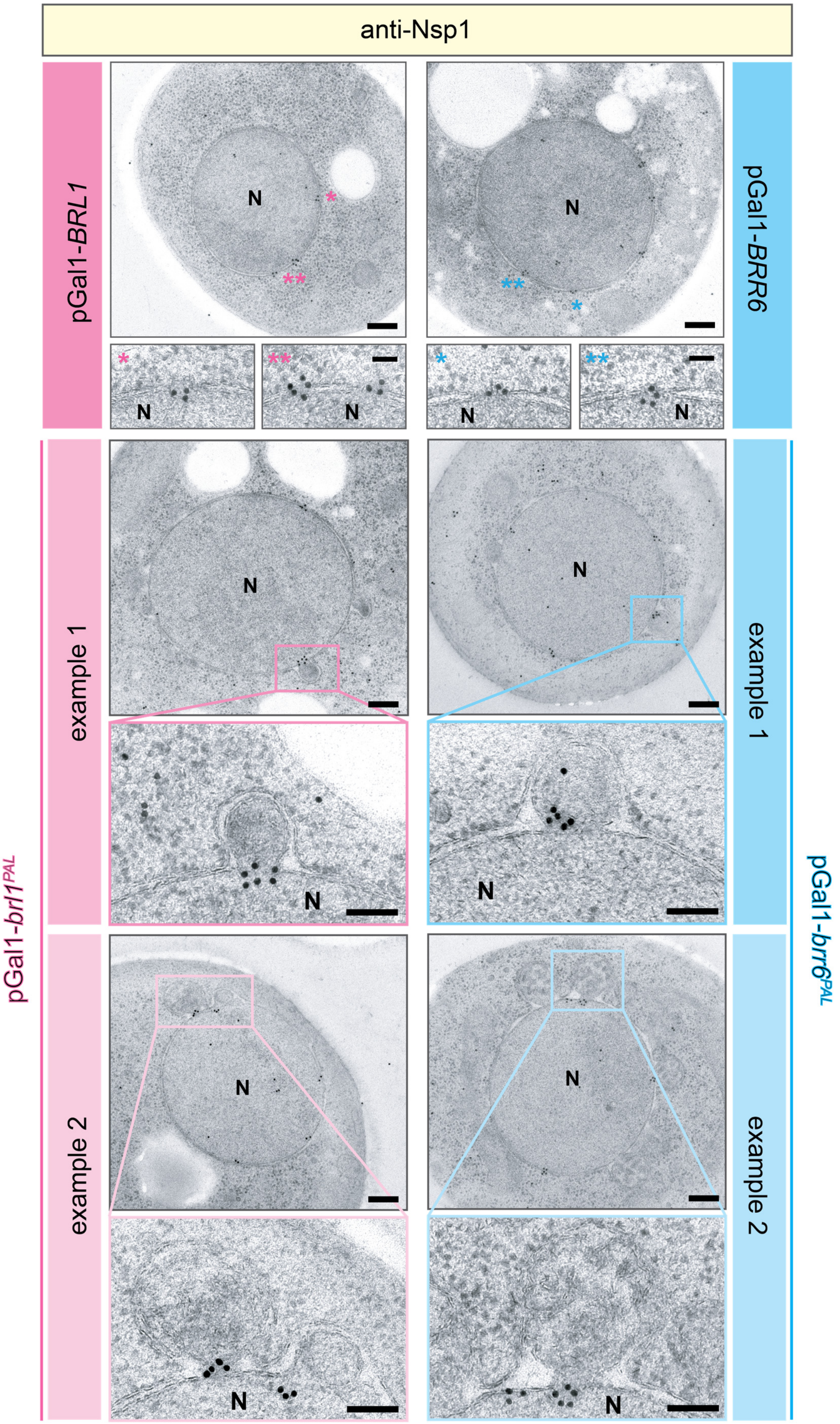
anti-Nsp1
pGal1-BRL1
pGal1-BRR6
pGal1-brl1PAL
pGal1-brr6PAL
example 1
example 2
N

Appendix Figure S4 - Nsp1 immuno-EM of pGal1-*BRR6* and pGal1-*brr6^{PAL}* cells. Extension of Figure 7.

Nsp1 immuno-EM of cells overexpressing pGal1-*BRL1,* pGal1-*brl1^{PAL},* pGal1-*BRR6* or pGal1-*brr6^{PAL}.* Size bars: 200 nm in the nuclear overviews; 100 nm and 50 nm in the small and large enlargements below, respectively. Note, the pink asterisks denote the corresponding enlargement areas under the overexpression condition of pGal1-*BRL1*, and the blue asterisks denote the corresponding enlargement areas under the overexpression condition of pGal1-*BRR6*. Abbreviation: N, nucleus.

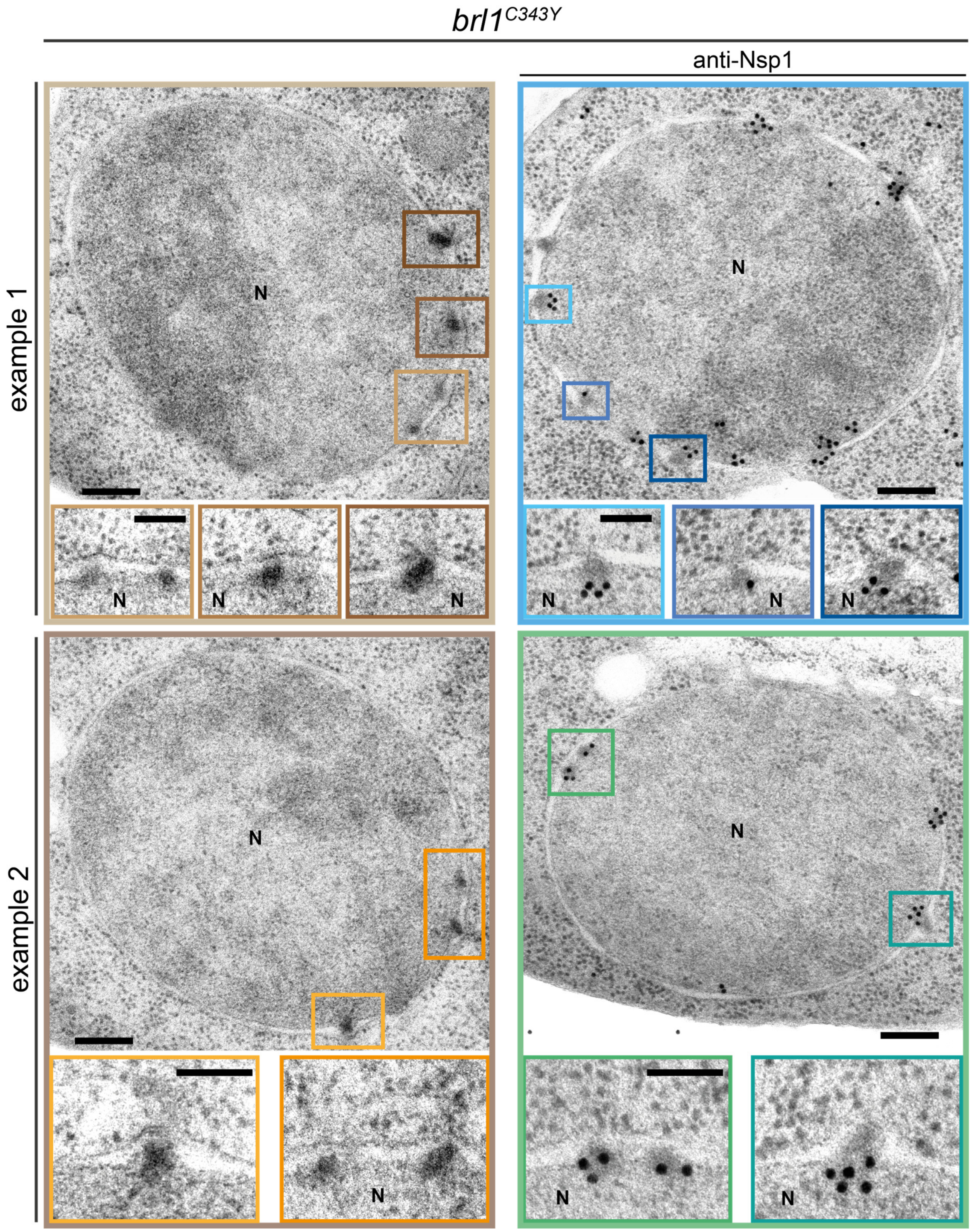

Appendix Figure S5 - *brl1^{C343Y}* mutant cells develop herniations. Extension of Figure 7.

Temperature-sensitive *brl1*^{C343Y} mutant cells (Vitale *et al.*, 2022) were incubated at 37ºC for 3 hours. EM analysis revealed the accumulation of herniations labeled with anti-Nsp1 antibodies. Asterisks indicated the enlarged regions. Size bars: 200 nm and 50 nm in the enlargements.

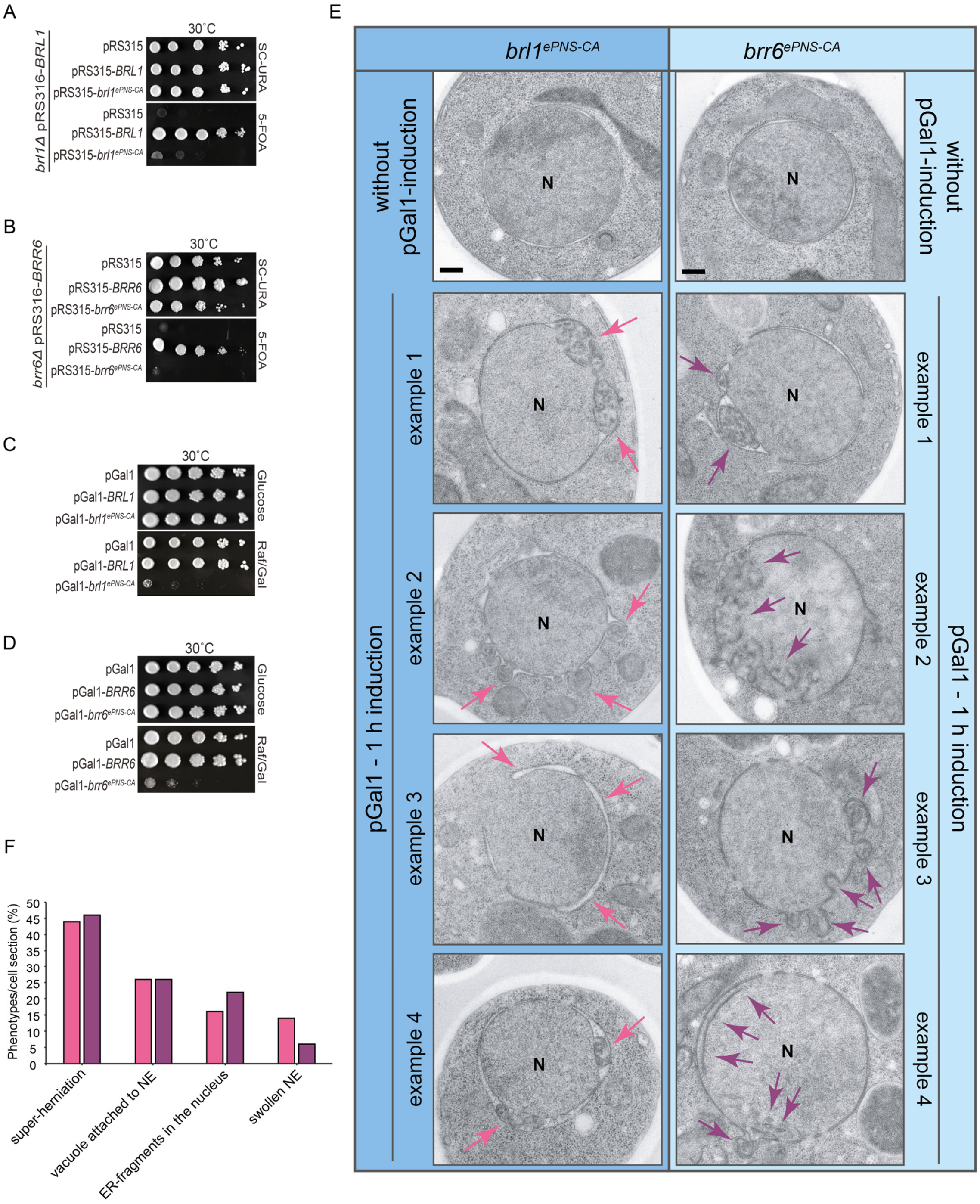

Appendix Figure S6 - Analysis of the pGal1-*brl1*$^{ePNS\text{-}CA}$ and pGal1-*brr6*$^{ePNS\text{-}CA}$ phenotypes. Extension of Figure 8.

(**A,B**) *brl1*$^{ePNS-CA}$ (A) and *brr6*$^{ePNS-CA}$ (B) constructs are non-functional in a plasmid shuffle assay at 30°C, whereas wild-type *BRL1* and *BRR6* are functional. Ten-fold serial dilutions; three independent experiments. **(C,D)** Overexpression of pGal1-*brl1*$^{ePNS-CA}$ (C) and pGal1-*brr6*$^{ePNS-CA}$ (D) is toxic to cells, while overexpression of wild-type *BRL1* and *BRR6* does not affect cell growth. Ten-fold serial dilutions; three independent experiments. (**E**) Analysis of the *brl1*$^{ePNS-CA}$ and *brr6*$^{ePNS-CA}$ phenotype by EM. pGal1-*brl1*$^{ePNS-CA}$ and pGal1-*brr6*$^{ePNS-CA}$ cells were grown for 1 hour with and without pGal1 induction. Cells were analyzed by thin section EM. Size bars: 500 nm. Note, the pink arrows indicate different phenotypes under the overexpression of pGal1-*brl1*$^{ePNS-CA}$, and the dark violet arrows indicate different phenotypes under the overexpression of pGal1-*brr6*$^{ePNS-CA}$. Abbreviation: N, nucleus. (**F**) Quantification of indicated phenotypes in pGal1-*brl1*$^{ePNS-CA}$ and pGal1-*brr6*$^{ePNS-CA}$ cells shown from (E). Total number of analyzed cells: 42 and 32 for pGal1-*brl1*$^{ePNS-CA}$ and pGal1-*brr6*$^{ePNS-CA}$, respectively in induced condition. These phenotypes were not observed in the controls without pGal1 induction.
