# Supplementary figures and images for: Multifunctional roles of Brl1-Brr6 in nuclear envelope fusion during nuclear pore complex biogenesis
Source: EMBO J. 2026 Feb 16;45(7):2370–99. doi: 10.1038/s44318-026-00718-y (PMC13043894; doi:10.1038/s44318-026-00718-y)

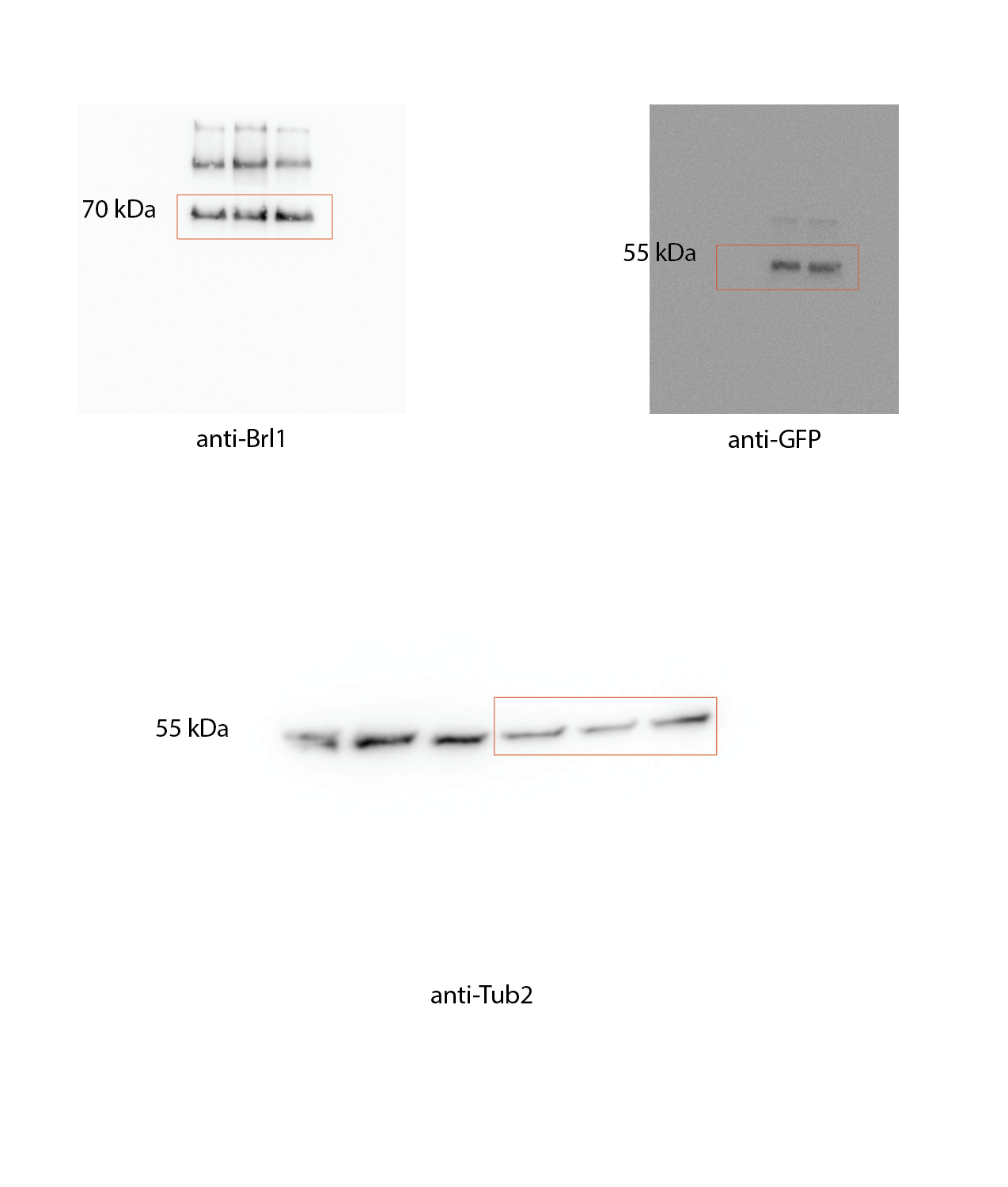

Supplement: Supplementary file 5 — Source data Fig. 5 [file 44318_2026_718_MOESM5_ESM.zip › Figure 5H/Figure 5H.tif]

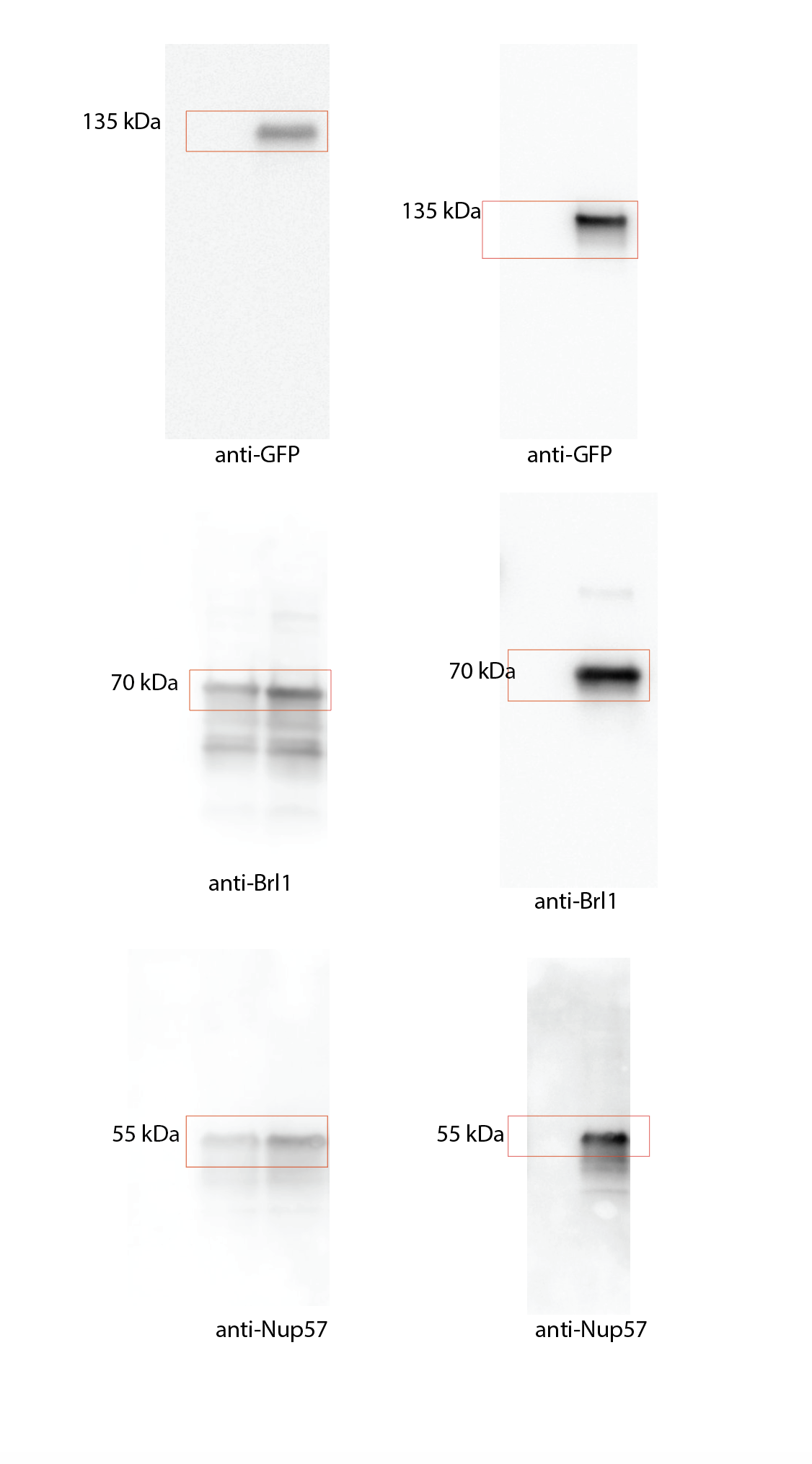

Supplement: Supplementary file 6 — Source data Fig. 6 [file 44318_2026_718_MOESM6_ESM.zip › Figure 6E/Figure 6E.tif]

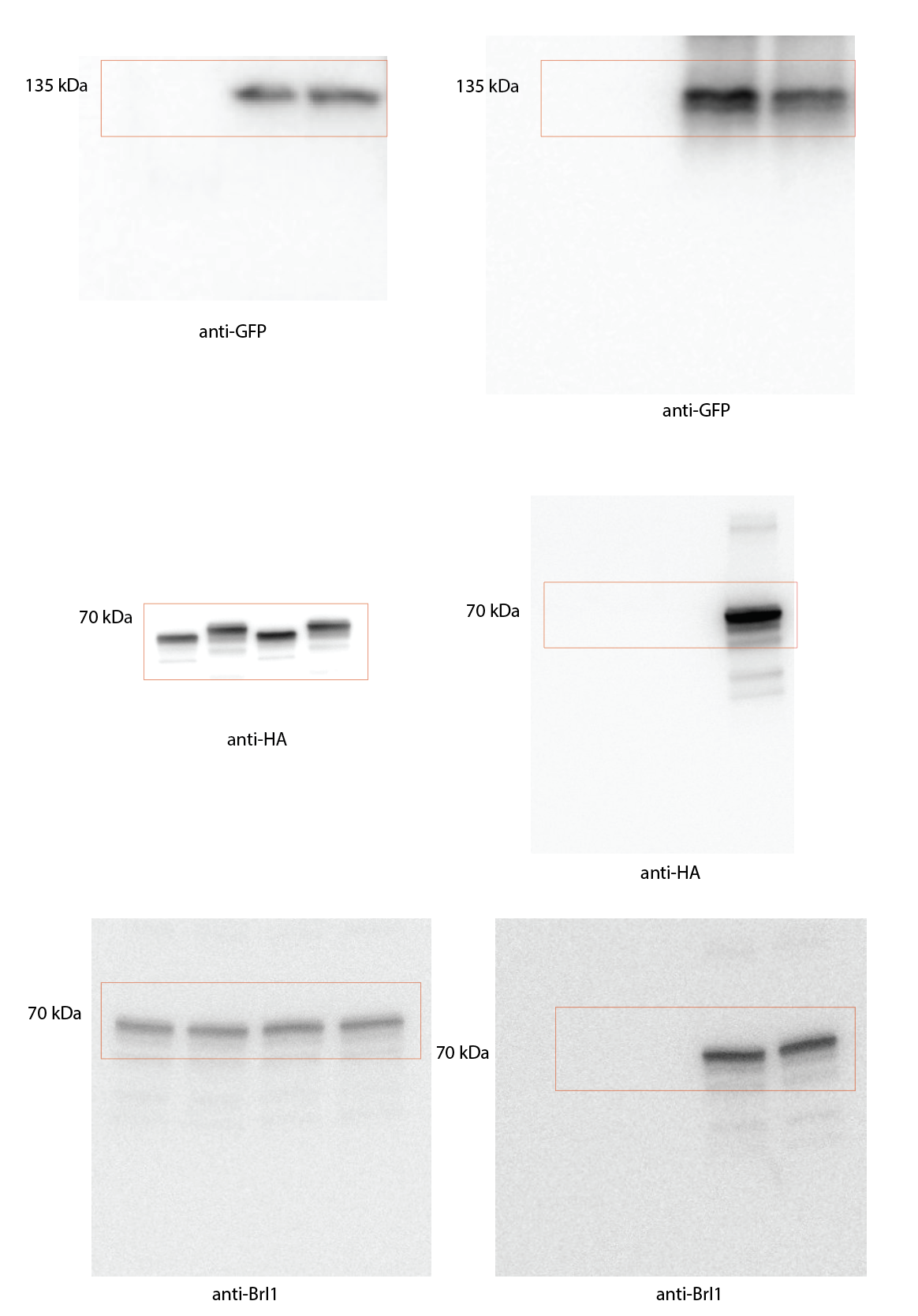

Supplement: Supplementary file 6 — Source data Fig. 6 [file 44318_2026_718_MOESM6_ESM.zip › Figure 6F/Figure 6F.tif]

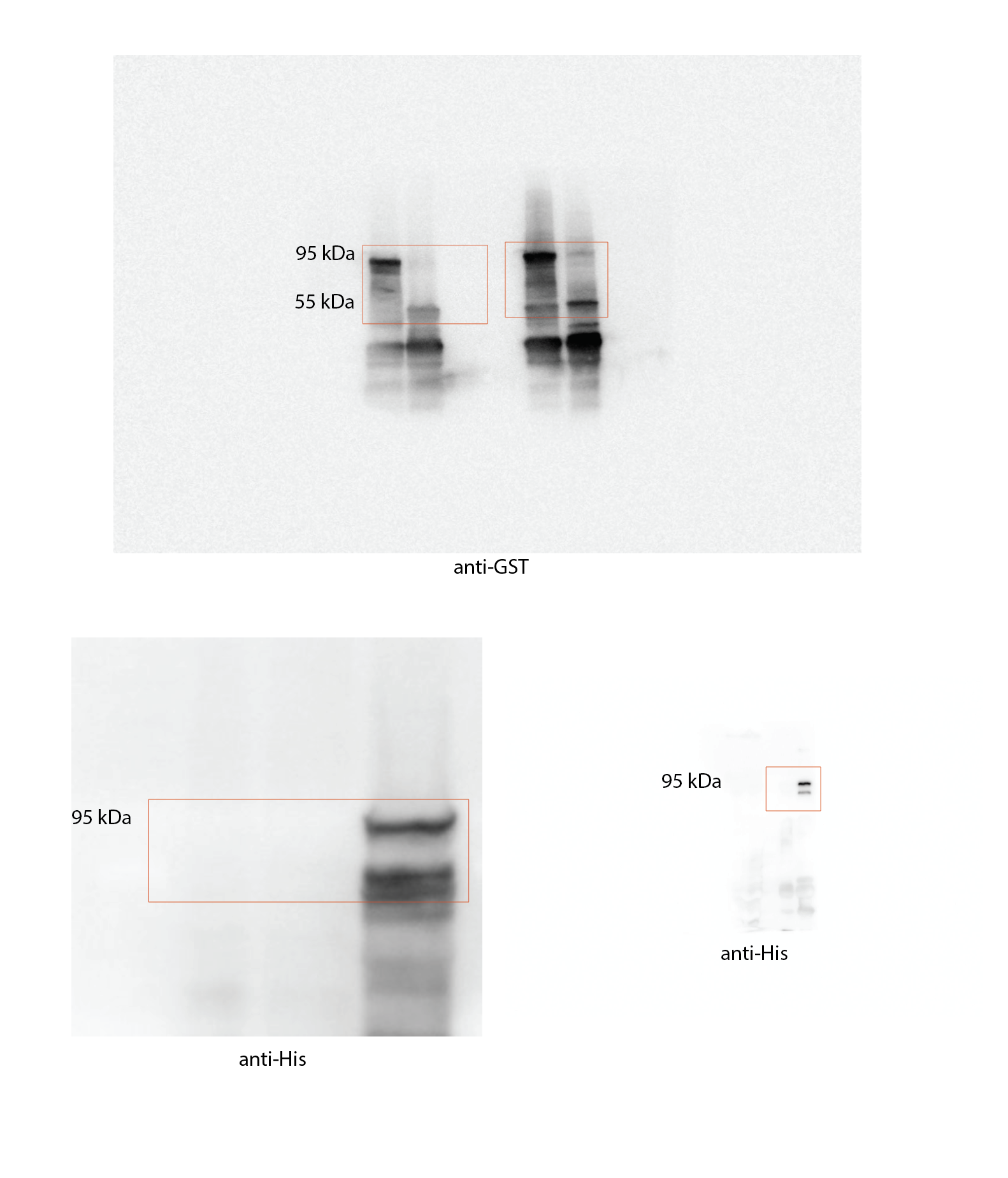

Supplement: Supplementary file 6 — Source data Fig. 6 [file 44318_2026_718_MOESM6_ESM.zip › Figure 6G/Figure 6G.tif]

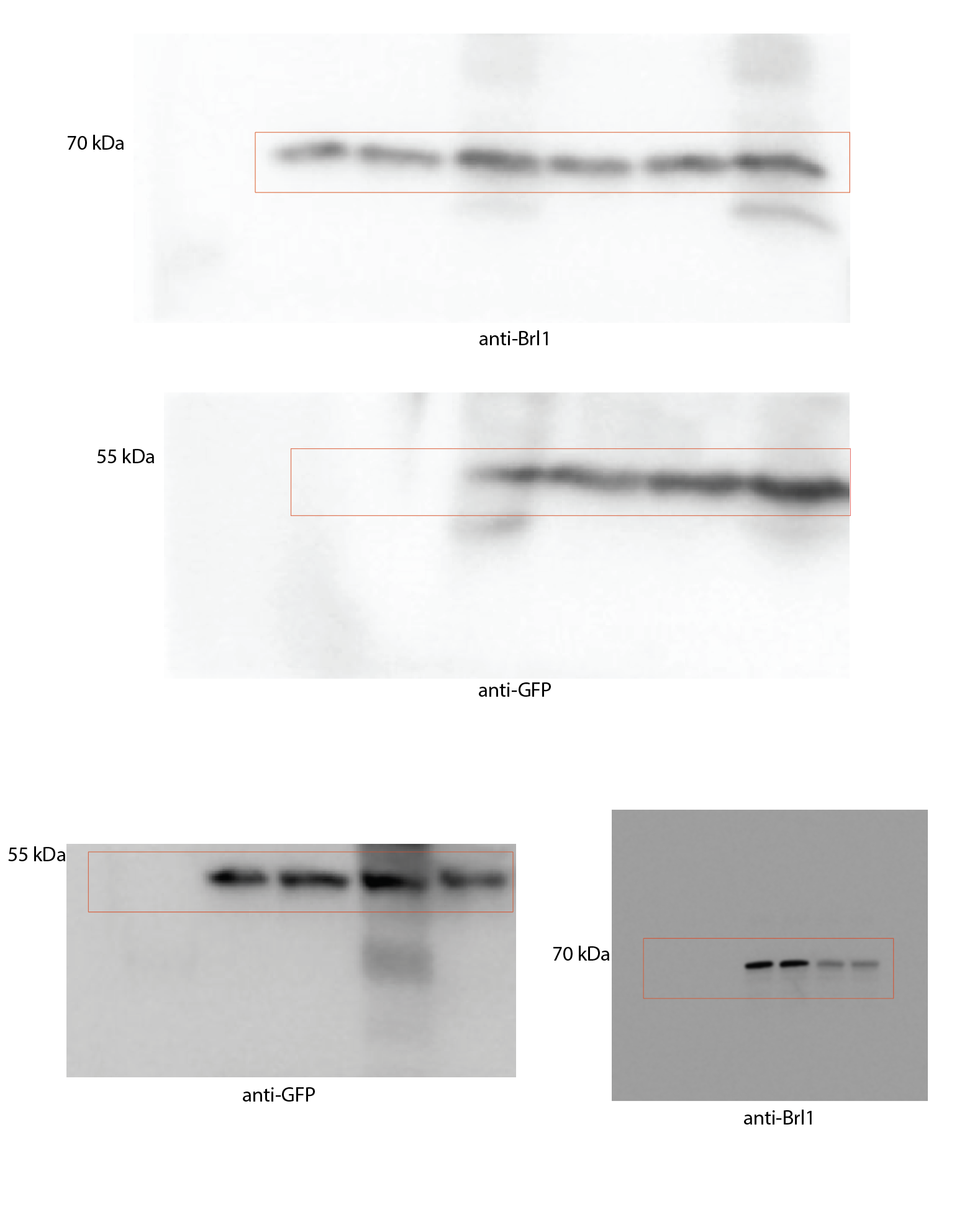

Supplement: Supplementary file 9 — Figure EV1 Source Data [file 44318_2026_718_MOESM9_ESM.zip › Figure EV1F/Figure EV1F.tif]
