# Supplementary material for: Multifunctional roles of Brl1-Brr6 in nuclear envelope fusion during nuclear pore complex biogenesis
Source: EMBO J. 2026 Feb 16;45(7):2370–99. doi: 10.1038/s44318-026-00718-y (PMC13043894; doi:10.1038/s44318-026-00718-y)
Supplement: Supplementary file 13 — Expanded View Figures [file 44318_2026_718_MOESM13_ESM.pdf]

Expanded View Figures

Figure EV1. AlphaFold predictions of Brr6-Brl1 interactions.

Extension of Fig. 1. (**A**) AlphaFold predicts full-lenh Brr6–Brl1 interactions mediated by the two AαH domains. The pTM and ipTM scores are indicated. (**B**) AlphaFold using the PNS domains predicts DAH/PAL region Brr6–Brl1 interactions. The pTM and ipTM scores are indicated. (**C**) AlphaFold predicts full-length brr6^{L145E}–Brl1 interactions via DAH/PAL but not the AαH domains. The pTM and ipTM scores are indicated. (**D**) AlphaFold predicts full-length Brr6–brl1^{F391E} interactions via DAH/PAL but not the AαH domains. The pTM and ipTM scores are indicated. (**E**) AlphaFold predicts full-length brr6^{L145E}–brl1^{F391E} interactions mediated by the two AαH domains. The pTM and pTM scores are indicated. (**F**) Immunoprecipitation experiment of brl1 and brr6 mutant proteins. Cells expressing the indicated *BRL1* and *BRR6* constructs were lysed and the Brl1-Brr6 interaction was analyzed by immunoprecipitation using GFP-binder. The immunoprecipitation was analyzed by immunoblotting with the indicated antibodies. Three independent experiments, one representative is shown. Source data are available online for this figure.

© The Author(s)

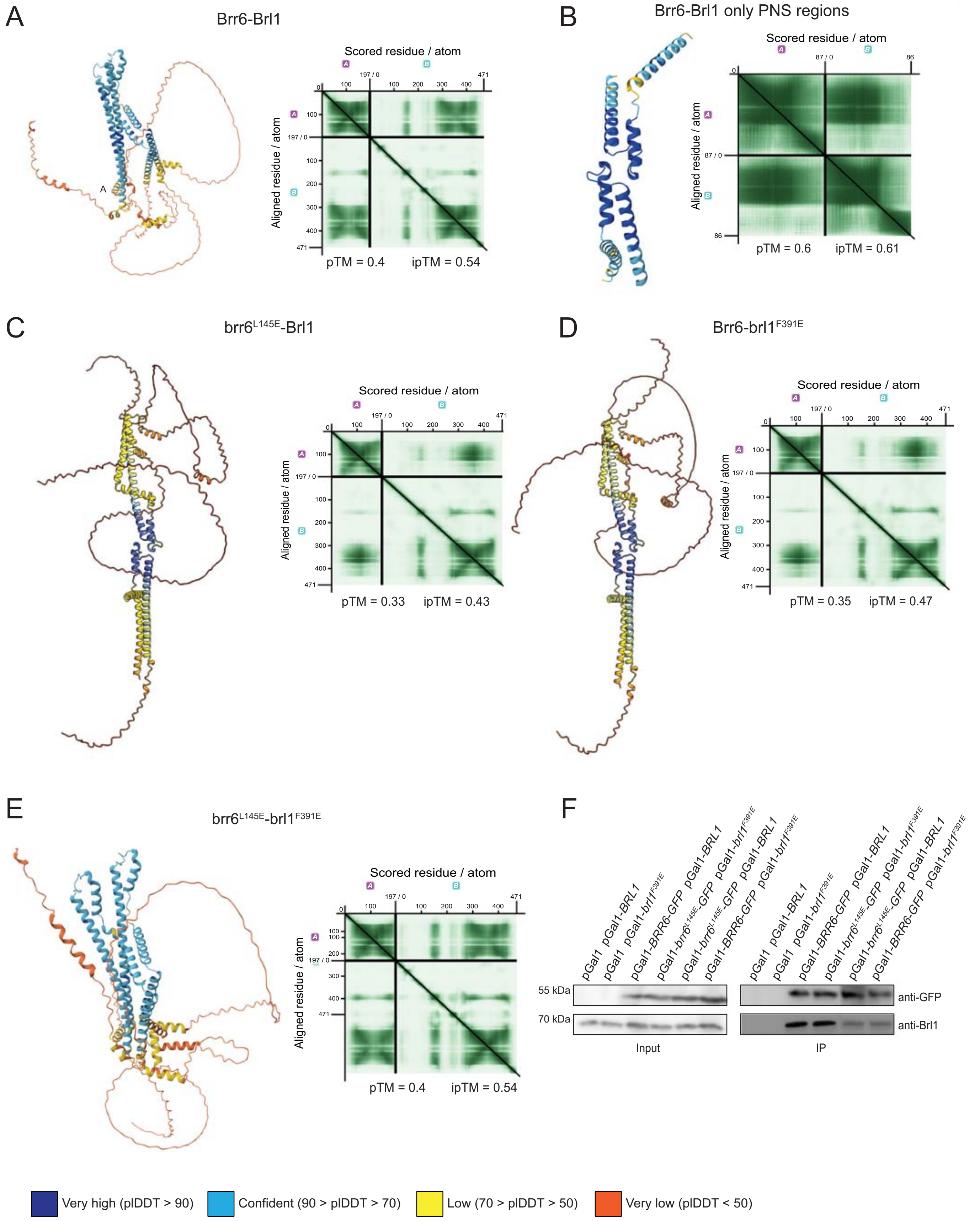
A
Brr6-Brl1
Scored residue / atom
Aligned residue / atom
pTM = 0.4
ipTM = 0.54
B
Brr6-Brl1 only PNS regions
pTM = 0.6
ipTM = 0.61
C
brr6L145E-Brl1
pTM = 0.33
ipTM = 0.43
D
Brr6-brl1F391E
pTM = 0.35
ipTM = 0.47
E
brr6L145E-brl1F391E
pTM = 0.4
ipTM = 0.54
F
pGal1 pGal1-BRL1
pGal1 pGal1-brl1F391E
pGal1-BRR6-GFP pGal1-BRL1
pGal1-brr6L145E-GFP pGal1-brl1F391E
pGal1-brr6L145E-GFP pGal1-BRL1
pGal1-BRR6-GFP pGal1-brl1F391E
55 kDa
70 kDa
anti-GFP
anti-Brl1
Input
IP
Very high (plDDT > 90)
Confident (90 > plDDT > 70)
Low (70 > plDDT > 50)
Very low (plDDT < 50)

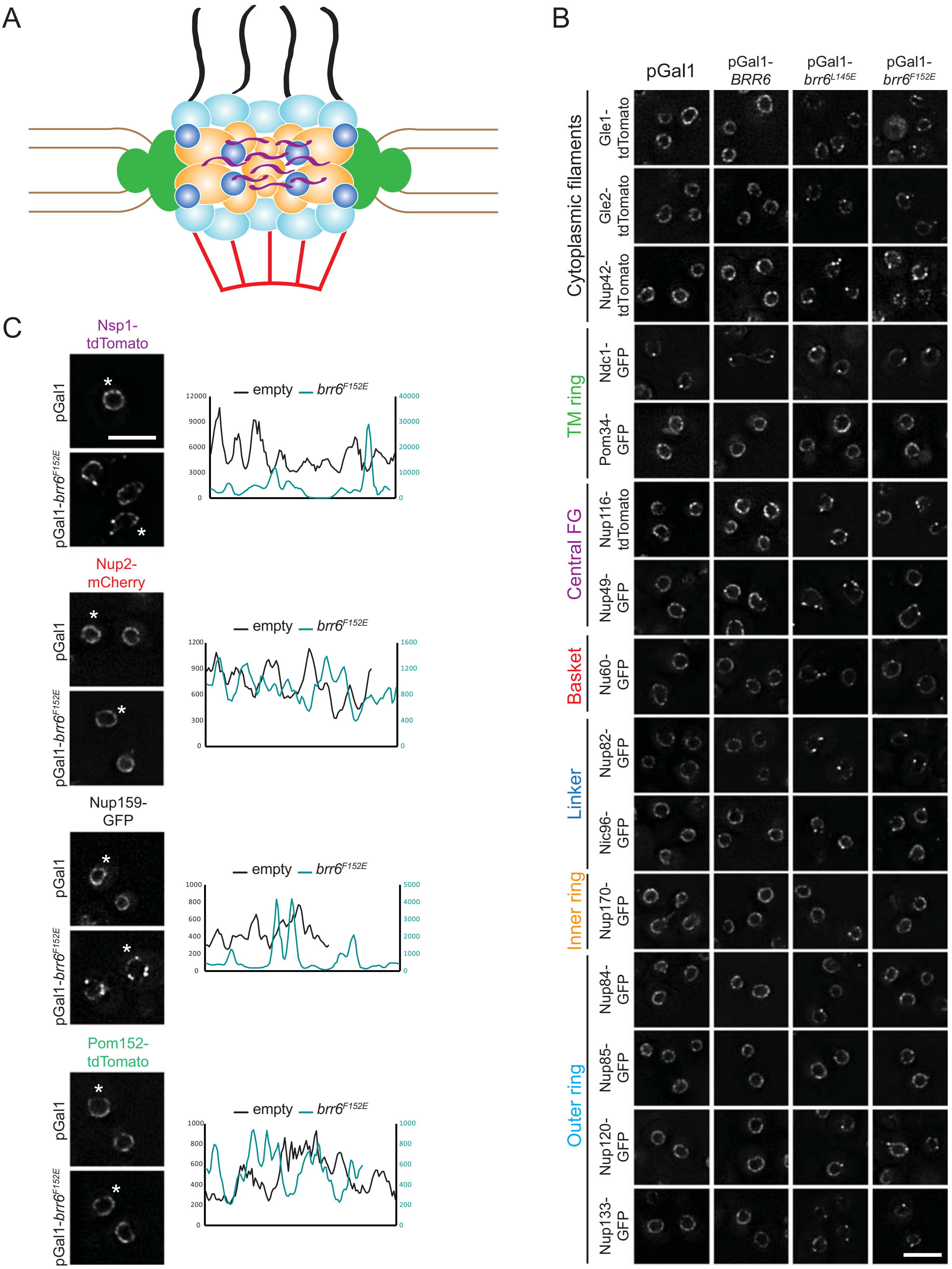
A
B
pGal1
pGal1-BRR6
pGal1-brr6L145E
pGal1-brr6F152E
Cytoplasmic filaments
Gle1-tdTomato
Gle2-tdTomato
Nup42-tdTomato
TM ring
Ndc1-GFP
Pom34-GFP
Central FG
Nup116-tdTomato
Nup49-GFP
Basket
Nu60-GFP
Linker
Nup82-GFP
Nic96-GFP
Inner ring
Nup170-GFP
Outer ring
Nup84-GFP
Nup85-GFP
Nup120-GFP
Nup133-GFP
C
Nsp1-tdTomato
pGal1
pGal1-brr6F152E
empty brr6F152E
Nup2-mCherry
Nup159-GFP
Pom152-tdTomato

 © The Author(s)

Figure EV2. Analysis of *brr6*^{L145E} and *brr6*^{F152E} overexpression phenotypes.

Extension of Fig. 3. (**A**) Schematic representation of the NPC with color-coded components: transmembrane ring (green), central FG-Nups (purple), nuclear basket Nups (red), linker Nups (blue), inner ring Nups (ochre), and outer ring Nups (light blue). (**B**) As in Fig. 3A using the indicated yeast strains expressing tdTomato- and GFP-tagged Nup constructs. Cells additionally carried pGal1, pGal1-*BRR6*, pGal1-*brr6*^{L145E}, or pGal1-*brr6*^{F152E}, which were overexpressed for 3 h. See Fig. 3B for quantification of the phenotypes. Size bar: 5 µm. (**C**) Overexpression of *brr6*^{F152E} alters NE localization of Nsp1 and Nup159, whereas Nup2 and Pom152 are only minimally affected. Line scans on the right show NE distribution of the indicated Nups in cells carrying either empty pGal1 or pGal1-*brr6*^{F152E} plasmids. The cell that was selected for line scan is indicated by an asterisk. Cells were incubated for 3 h in raffinose/galactose medium to induce expression from the pGal1 promoter. Note, the *NSP1-tdTomato* pGal1-*brr6*^{F152E} cells (top) are identical to the cells in Fig. 3A (first column). Size bar: 5 µm. Source data are available online for this figure.

© The Author(s)

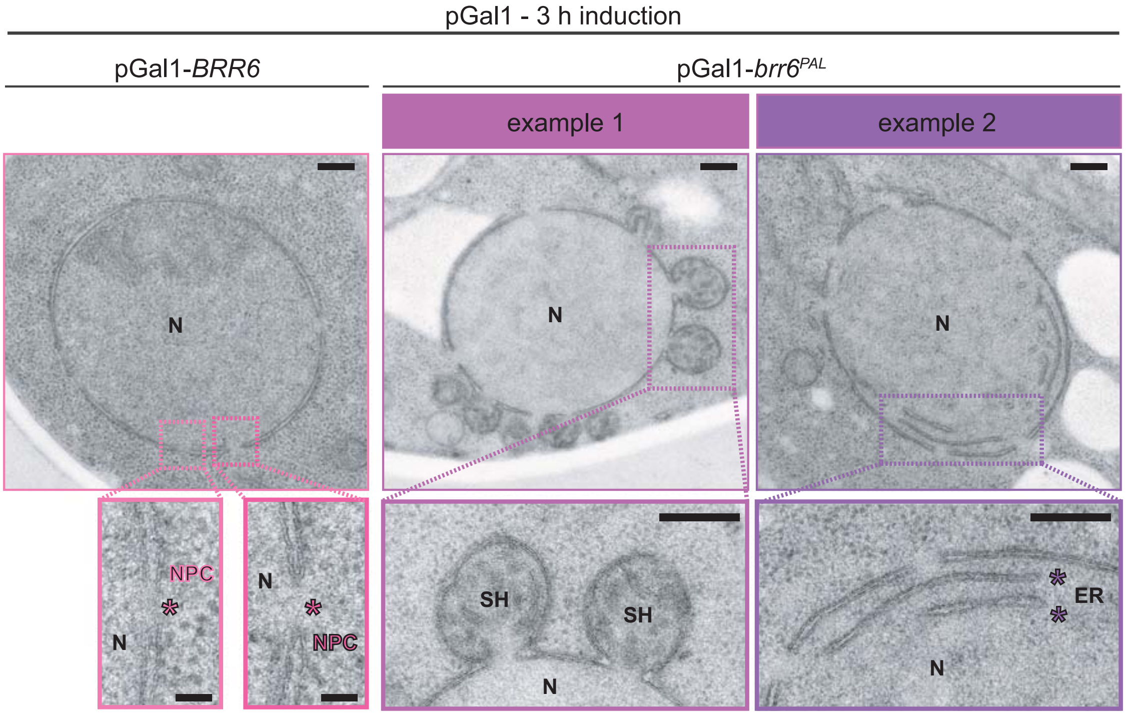

Figure EV3. Phenotypes of *brr6*^{PAL} mutant overexpression.

Extension of Fig. 7. EM analysis of cells overexpressing pGal1-*BRR6* or pGal1-*brr6*^{PAL} using the same conditions as in Fig. 7J revealed accumulation of superherniations (SH) at the NE. The pGal1-*BRL1* or pGal1-*brl1*^{PAL} data are shown in Fig. 7J. Representative EM images are shown. Size Bars: 200 nm in the row showing nuclei; 50 nm in the enlargements with the NPCs; 200 nm in the enlargements showing the superherniations (SH) or ER inside the nucleus (example 2; purple asterisk). The pink asterisks highlight NPCs in pGal1-*BRR6* cells. ER endoplasmic reticulum, N nucleus, NPC nuclear pore complex, SH superherniation. Source data are available online for this figure.

 © The Author(s)

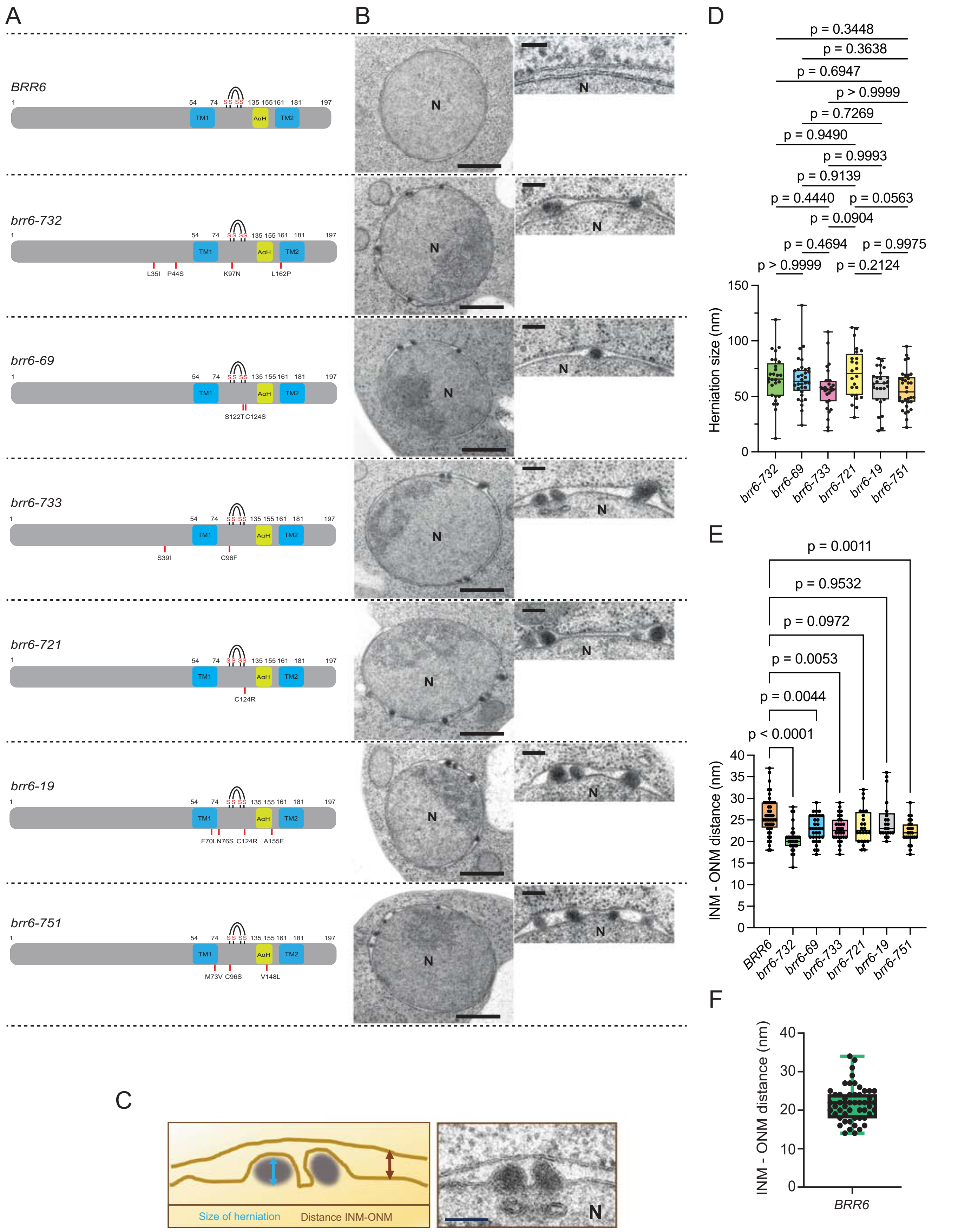
A
B
D
BRR6
brr6-732
L35I P44S K97N L162P
brr6-69
S122TC124S
brr6-733
S39I C96F
brr6-721
C124R
brr6-19
F70LN76S C124R A155E
brr6-751
M73V C96S V148L
TM1
AαH
TM2
N
p = 0.3448
p = 0.3638
p = 0.6947
p > 0.9999
p = 0.7269
p = 0.9490
p = 0.9993
p = 0.9139
p = 0.4440
p = 0.0563
p = 0.0904
p = 0.4694
p = 0.9975
p > 0.9999
p = 0.2124
Herniation size (nm)
E
p = 0.0011
p = 0.9532
p = 0.0972
p = 0.0053
p = 0.0044
p < 0.0001
INM - ONM distance (nm)
C
Size of herniation
Distance INM-ONM
F

Figure EV4. Brr6 also functions in INM/ONM fusion during NPC biogenesis.

Extension of Fig. 7. (**A**) Overview of conditional lethal *brr6(ts)* alleles generated by mutagenic PCR (Zhang et al, 2018). The resulting *brr6(ts)* alleles were verified by sequencing and integrated into the endogenous *BRR6* locus using a pop-in pop-out strategy (Rothstein, 1991). The amino acid substitutions in the various *brr6(ts)* alleles are indicated. TM, AαH, and the four cysteines involved in disulfide bridge formation are also marked. Note, these mutants were previously only analyzed in the context of benzyl alcohol sensitivity (Zhang et al, 2018). (**B**) The wild-type and *brr6(ts)* mutants were shifted to 37 °C for 3 h, and their phenotype was analyzed by thin-section EM. All *brr6(ts)* mutants accumulated herniations at the NE. N nucleus. Size bar: 500 nm; magnified inset: 50 nm. (**C**) Scheme for the analysis of wild-type and *brr6(ts)* mutant cells. Size bar: 100 nm. Note that the micrograph next to the schematic is a re-display of a section from (**B**) for illustrative purposes. (**D**, **E**) Herniation size (**D**) and random INM–ONM distance (**E**) away from herniations were measured from EM sections in (**B**). In wild-type cells, INM–ONM distances were measured randomly away from NPCs. (**D**) The size of 24, 32, 28, 24, 26 and 33 herniations from 11, 10, 10, 8, 7, 8 cell sections of *brr6-732*, *brr6-69*, *brr6-733, brr6-721*, *brr6-19* and *brr6-751* cells was measured. (**E**) The INM–ONM distance from 16, 11, 11, 10, 8, 7 and 8 cell sections with three measurement per section was determined. Statistical test: One-way ANOVA. Box and Whisker plots shows median with interquartile range. $P = 2.2E\text{-}8$ for the statistical significance between *BRR6* and *brr6-732* condition (**E**). (**F**) The distance between the INM and ONM in wild-type cells grown at 30 °C was measured by EM. INM–ONM distances were measured randomly away from NPCs. Ultrathin sections from 16 cells with four measurement per section were analyzed, revealing an average INM–ONM spacing of 22 nm. Data shows median with interquartile range. Source data are available online for this figure.

 © The Author(s)

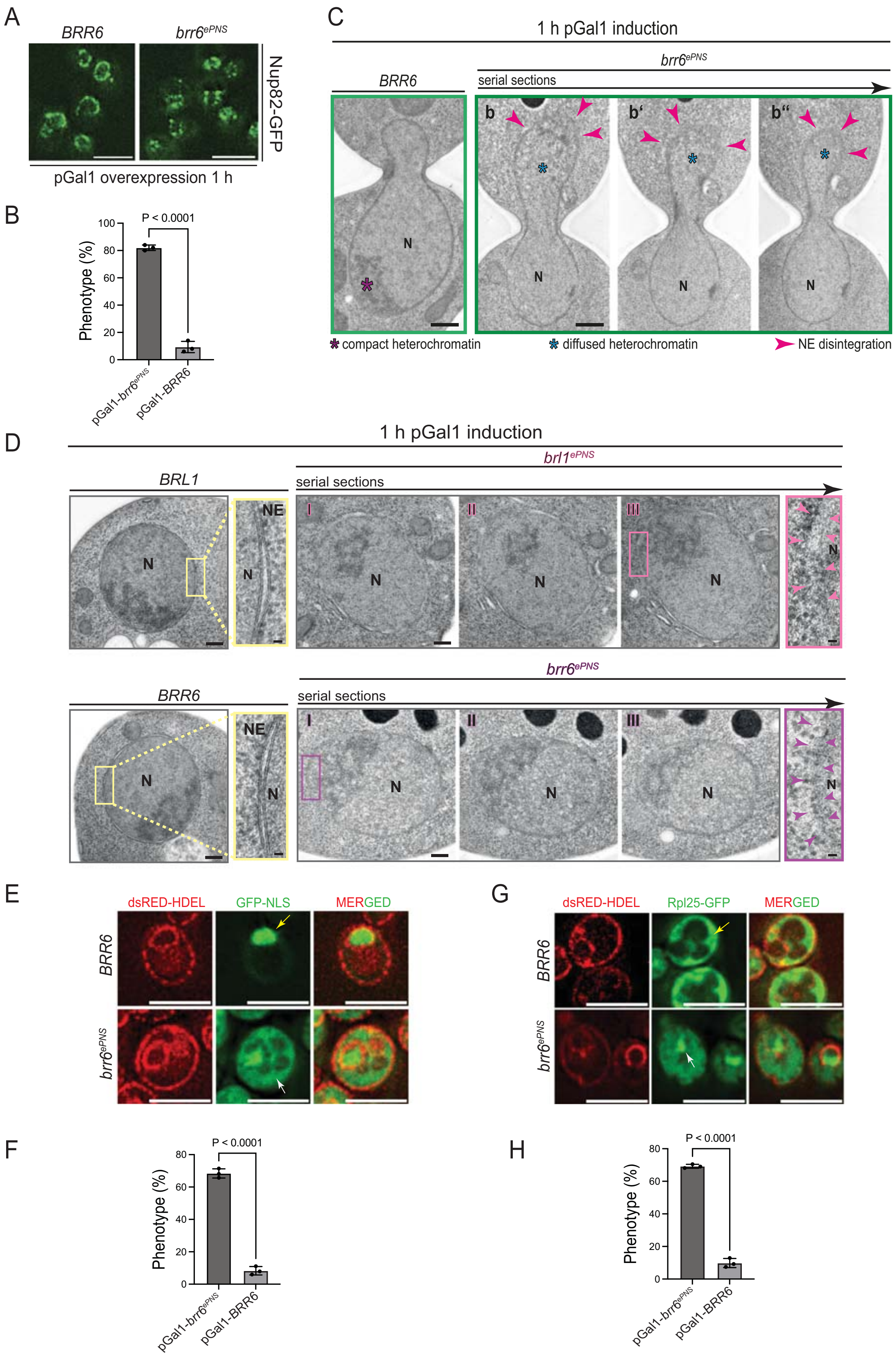
A
BRR6
brr6ePNS
Nup82-GFP
pGal1 overexpression 1 h
B
P < 0.0001
Phenotype (%)
pGal1-brr6ePNS
pGal1-BRR6
C
1 h pGal1 induction
brr6ePNS
BRR6
serial sections
compact heterochromatin
diffused heterochromatin
NE disintegration
D
1 h pGal1 induction
brl1ePNS
BRL1
serial sections
brr6ePNS
BRR6
serial sections
E
dsRED-HDEL
GFP-NLS
MERGED
BRR6
brr6ePNS
G
dsRED-HDEL
Rpl25-GFP
MERGED
BRR6
brr6ePNS
F
P < 0.0001
Phenotype (%)
pGal1-brr6ePNS
pGal1-BRR6
H
P < 0.0001
Phenotype (%)
pGal1-brr6ePNS
pGal1-BRR6

Figure EV5. Phenotypes of *brr6*^{ePNS} overexpression.

Extension of Fig. 8. (**A**) Analysis of pGal1-*BRR6* and pGal1-*brr6*^{ePNS} cells carrying *NUP82-GFP*. Localization of Nup82-GFP was analyzed by fluorescence microscopy. Three independent experiments. Size bars: 5 µm. Note, the corresponding experiment with pGal1-*BRL1 and* pGal1-*brl1*^{ePNS} is shown in Fig. 8G. (**B**) Quantification of (A). Total number of analyzed cells: 150 each for both pGal1-*BRR6 and* pGal1-*brr6*^{ePNS}; Statistical test: unpaired two-tailed *t* test. Data represent the mean ± SD from three independent experiments; ($P = 1.1E\text{-}5$). (**C**) EM analysis of cells expressing pGal1-*BRR6* or pGal1-*brr6*^{ePNS} after 1 h of galactose induction. Panels b–b″ show 80 nm serial sections of the same anaphase cell. Red arrowheads indicate regions of the NE undergoing disintegration. Purple asterisk marks compact chromatin; blue asterisks denote decondensed chromatin adjacent to disintegrating NE regions. N nucleus. Size bars: 500 nm. The corresponding experiment with pGal1-*BRL1* and pGal1-*brl1*^{ePNS} is shown in Fig. 8I. (**D**) Shows EM analysis of pGal1-*BRL1*, pGal1-*brl1*^{ePNS}, pGal1-*BRR6* and pGal1-*brr6*^{ePNS} cells in interphase. I-III indicates serial sections. Arrowheads in enlargements show disintegrated NE. Size bars: 500 nm; enlargements 25 nm. N nucleus, NE nuclear envelope. (**E**) pGal1-*brr6*^{ePNS} overexpression affects localization of GFP–NLS in the nucleus. *GFP-NLS dsRED-HDEL* cells with pGal1-*BRL1* or pGal1-*brl1*^{ePNS} were incubated for 1 h with galactose. Localization of GFP–NLS was analyzed by fluorescence microscopy. Size bar: 5 µm. The yellow arrow indicates GFP–NLS in the nucleus; the white arrow NLS-GFP in the cytoplasm. (**F**) Cells from (**E**) were quantified for the localization of GFP–NLS. Three independent experiments with total number of analyzed cells 218 and 216 for pGal1-*BRR6 and* pGal1-*brr6*^{ePNS}, respectively. Statistical test: unpaired two-tailed *t* test. Data shows mean with SD; ($P = 1.13E\text{-}5$). (**G**) As (**E**) but with *dsRED-HDEL RPL25-GFP* cells. The yellow arrow indicates Rpl25–GFP in the cytoplasm. The white arrow indicates Rpl25–GFP in the nucleus. Size bars: 5 µm. (**H**) Cells from (**G**) were quantified for the localization of Rpl25–GFP. Three independent experiments with total number of analyzed cells 281 and 311 for pGal1-*BRR6* and pGal1-*brr6*^{ePNS}, respectively. Statistical test: unpaired two-tailed *t* test. Data shows mean with SD ($P = 4.5E\text{-}6$). Source data are available online for this figure.

 © The Author(s)
